# Expression and role of HSPA5 in environmental toxin-induced neurological disorders

**DOI:** 10.3389/fnmol.2026.1964213

**Published:** 2026-09-21

**Authors:** Yurong Wang, Yang Lv, Ruidong Zhao, Xin Guo, Jing Gao, Tong Wang, Dongmei Yao, Xin Li, Bingchun Liu, Jianlong Yuan

**Affiliations:** 1 Department of Clinical Laboratory, Affiliated Hospital of Inner Mongolia Medical University, Hohhot, China; 2 Central Laboratory of Organ Transplantation, Affiliated Hospital of Inner Mongolia Medical University, Hohhot, China

**Keywords:** cellular damage, environmental toxins, ER stress, HSPA5, neurodegenerative diseases

## Abstract

HSPA5 is a member of the HSP70 family of heat shock proteins (HSPs) and functions as a chaperone protein within the endoplasmic reticulum (ER). It plays a role in maintaining ER homeostasis, assists in correct protein folding, and triggers the unfolded protein response. Environmental toxins–such as heavy metals, microplastics (MPs), and air pollutants–can cause cellular damage. These toxins can trigger ER stress, resulting in the buildup of proteins that are misfolded or not properly folded, which in turn upregulates HSPA5 expression. Moderate activation of HSPA5 serves as a protective mechanism aimed at restoring protein homeostasis and promoting cell survival; however, sustained and severe ER stress may cause HSPA5 to malfunction, ultimately leading to the activation of the apoptotic pathway and resulting in cell death. Furthermore, HSPA5-mediated mechanisms are closely associated with the prodromal initiation and subsequent escalation of neurodegenerative syndromes. This article focuses on the role of HSPA5 in environmental toxin-induced neurotoxicity and neurodegenerative diseases, with the aim of investigating changes in HSPA5 expression and its regulatory functions in neurotoxicity, thereby providing new strategies for the study of environment-related neurodegenerative diseases.

## Graphical Abstract

**Figure:**
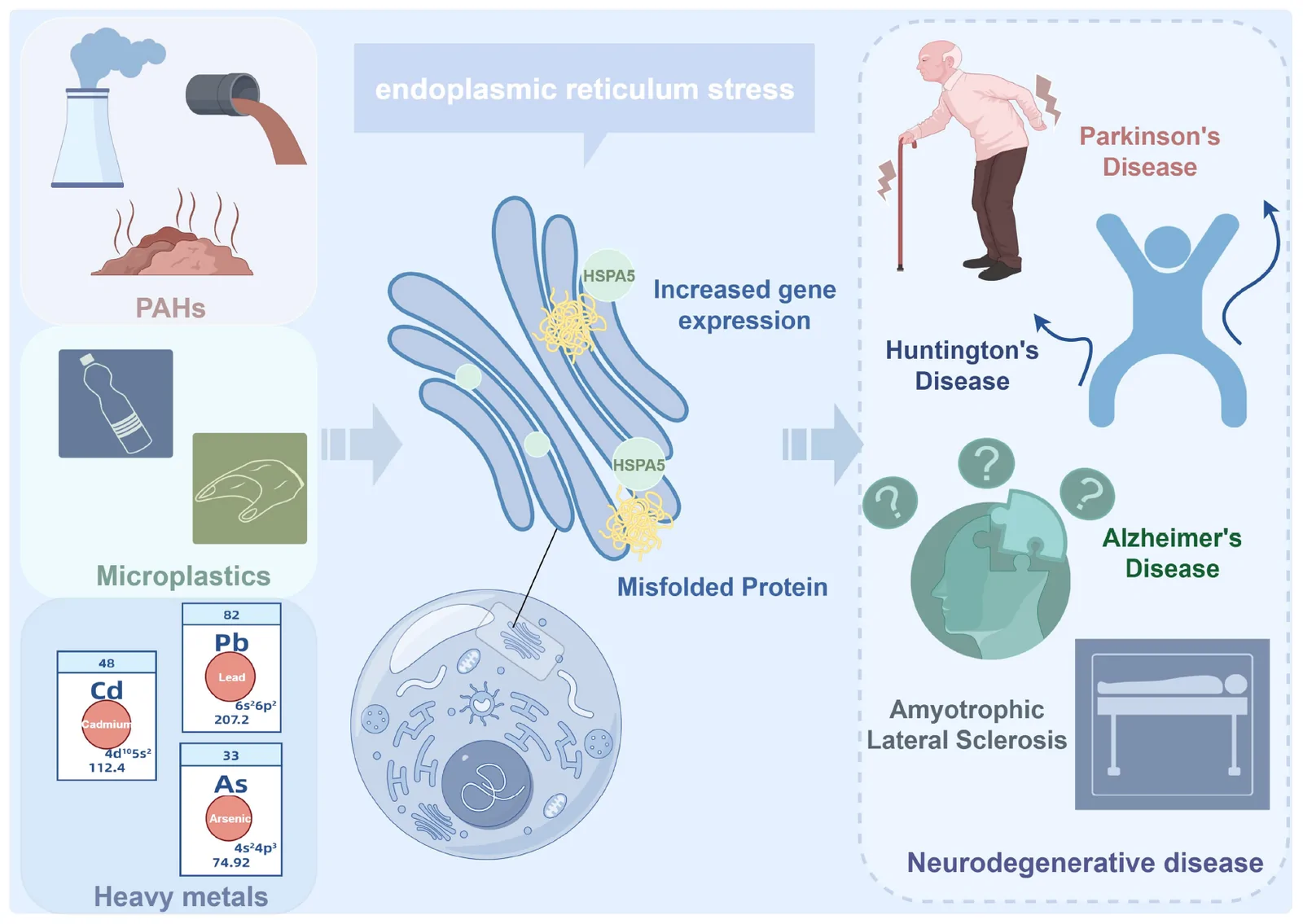
Endoplasmic reticulum stress induced by environmental toxins and neurological disorders.

## 1 Introduction

Encoded by the Hspa5 gene, the HSPA5 protein, also known as glucose-regulated protein 78 (GRP78) or immunoglobulin-binding protein (BiP), is a typical paralog within the HSP70 chaperone family (Xiao et al., 2023). As a chaperone protein, it serves as a principal mediator of the maintenance of endoplasmic reticulum (ER) homeostasis. The ER is a continuous membrane-bound organelle composed of an anastomosing system of sheet-like cisternae juxtaposed to the nuclear envelope that performs multiple critical homeostatic duties within the cell. It participates in the synthesis of cholesterol and phospholipids within the ER lumen, providing essential components for cell membrane formation; simultaneously, it is responsible for the storage and release of calcium ions to maintain intracellular calcium homeostasis (Zhang J. et al., 2022). Furthermore, the ER collaboratively regulates secretory protein synthesis, proper tertiary assembly, and vesicular conveyance. Given that properly folded proteins are fundamental to cellular life, the ER critically orchestrates the maintenance of protein conformational fidelity (Chen L. W. et al., 2021). Under physiological conditions, HSPA5 interacts with ER stress sensor proteins and maintains them in an inactive state. ER stress occurs when the ER cannot mediate proper protein folding, resulting in the accumulation of misfolded proteins in the ER lumen and causing HSPA5 to detach from sensor proteins, which activates the unfolded protein response (UPR) (Šereš et al., 2020). The UPR seeks to reestablish ER homeostasis through mechanisms such as the promotion of degradation of aberrantly folded proteins, the suppression of global protein translation, and the upregulation of the expression of chaperone proteins. Nonetheless, if ER stress continues and remains unresolved, the UPR shifts toward a proinflammatory response, which may ultimately induce cell death (Zhang J. et al., 2022).

Environmental toxins, through various pathways, cause multiple and far-reaching disruptions to natural ecosystems and human well-being (Piscopo et al., 2024). Among these, there is a strong association between HSPA5 and exposure to environmental toxins, notably polycyclic aromatic hydrocarbons (PAHs), heavy metals, and microplastics (MPs). PAHs are a widespread group of organic pollutants produced mainly by the incomplete burning or pyrolysis of materials such as fossil fuels, biomass, vehicle emissions, domestic heating and cooking, and tobacco smoke. They are widely present in the air, water, and soil and pose a significant threat to public health (Liu B. et al., 2025). PAHs can induce oxidative stress in cells, leading to an increase in free radicals. Different environmental toxins can upset the equilibrium of the ER and initiate a stress response (Ekundayo et al., 2024). MPs are a type of pollutant characterized by plastic pieces less than 5 millimeters wide. They remain in the environment, are difficult to break down, and are readily taken up by living organisms. During the immune response, there is a notable increase in the reactive oxygen species (ROS) level, which accumulates and causes oxidative stress and cellular injury (Prüst et al., 2020). Heavy metals in polluted air, water, and soil can infiltrate the human body through different pathways, leading to their accumulation in significant amounts within human tissues (Notariale et al., 2021). Arsenic, cadmium, and lead are harmful heavy metals that the human body struggles to process and eliminate, leading to their accumulation in critical organs such as the brain, heart, and kidneys. These metals disrupt normal bodily functions (Jannetto and Cowl, 2023).

Recently, interest in the function of HSPA5 in cellular and nervous system activities has increased. Under mild stress conditions, the expression of HSPA5 is crucial. It temporarily triggers the UPR to postpone apoptosis. If ER stress becomes excessive or prolonged, the positive and protective effects of UPR signaling are lost, leading to apoptosis. This makes HSPA5 an extremely appealing subject for research. Consequently, this review seeks to clarify the function of HSPA5 in cells and its involvement in neurological disorders. It provides a theoretical framework for protecting cells and developing new therapies to intervene in the progression of neurodegenerative diseases.

## 2 HSPA5

### 2.1 HSPA5 and heat shock proteins

The heat shock response (HSR) is a highly conserved cellular protective mechanism that defends against damage caused by various stressors, chemical toxins, ultraviolet radiation, and oxidants (Singh M. K. et al., 2024). This response activates a specific set of genes and their corresponding protein products, collectively known as heat shock proteins (HSPs). The HSR includes constituents of the small HSP family, which collectively maintain protein homeostasis (Davoudi et al., 2022). HSPs are found in bacterial, plant, and animal cells, and their primary function is to monitor and regulate cellular processes (Zhang and Yu, 2022). Pathological conditions such as oxidative stress, infection, and inflammation can induce the expression of HSPs (Uyar et al., 2022). HSPs are key molecules involved in the response to various forms of cellular stress; they can fold newly synthesized peptide chains, causing misfolded proteins to unfold and refold, degrading abnormal proteins, and transporting functional proteins (Guan et al., 2022; Marques et al., 2021; Zuo et al., 2024). HSPA5, a member of the HSP70 family, is found in the ER lumen and is vital to human health because of its ability to change its function through posttranslational modifications (Rehati et al., 2023).

### 2.2 HSPA5 and molecular chaperones

Chaperones are crucial for maintaining protein homeostasis, which is vital for cell integrity. Chaperones protect the proteome by forming complex, collaborative networks (Dawar et al., 2024). Various proteins within this network work synergistically to perform specific chaperone functions, including facilitating the folding of proteins into their native conformation, preventing misfolding, and degrading or isolating potentially toxic oligomers (Karamanos and Clore, 2022). HSPs are an important class of chaperones (Bai et al., 2023). In addition to participating in the folding of newly synthesized proteins, HSPs also mediate multiple key processes involved in proteostasis, including the biogenesis of multimeric assemblies, intracellular trafficking, and proteolytic turnover, as well as the disassembly and conformational restoration of stress-induced protein aggregates (Zhao Z. et al., 2024). Various stressors can induce regulatory responses, upregulating the expression levels of specific HSPs, thereby reinforcing the mechanisms involved in maintaining protein homeostasis (Mohanty et al., 2022). HSPA5, a member of the HSP70 family (Jiang J. et al., 2022), is the ER homolog of HSP70 and consists of an ATPase domain, a peptide-binding domain, and an ER retention signal (KDEL). Unlike cytoplasmic HSP members, HSPA5 contains a signal sequence that directs its localization to the ER (Deng et al., 2022). As an important chaperone in the ER, HSPA5 is crucial for the folding and processing of proteins that are destined for membrane formation or secretion (Elshemey et al., 2022; Liu Z. et al., 2023).

### 2.3 HSPA5 and the ER

#### 2.3.1 ER

The ER is a continuous membrane system composed of an interconnected network of tubules that extends throughout the cytoplasm (Gao et al., 2022). It can be classified into smooth ER and rough ER. The rough ER has ribosomes attached to its surface and is involved in protein synthesis, processing, and transport (Guan et al., 2024). Many proteins are conveyed to the ER (Neal et al., 2020). In the ER, these proteins are initially folded and assembled, after which they are transported to downstream secretory compartments. To carry out their essential functions, proteins need to maintain their proper folded structure and functional activity. The ER quality control (ERQC) pathway mainly ensures the integrity of these proteins (Wiseman et al., 2022). This pathway includes chaperones, folding enzymes, degradation factors, and factors that regulate the transport of mature proteins from the ER to the Golgi apparatus (Mesgarzadeh et al., 2022). As new proteins enter the ER lumen, molecular chaperones and folding enzymes facilitate their maturation into active forms (Woodward et al., 2020). Afterward, proteins that are properly folded are moved to the Golgi apparatus, while those that are misfolded and do not pass through the apparatus are marked. The ER-associated degradation (ERAD) pathway clears them via the proteasome (Smith et al., 2021), or they are degraded by lysosomes through ER autophagy (Reggiori and Molinari, 2022). This process promotes the release of properly folded proteins while preventing the release of improperly folded proteins (Chai et al., 2022).

#### 2.3.2 ER stress and the misfolded protein response

Various forms of cellular stress can disrupt the dynamic equilibrium between chaperone capacity and folding load, leading to the disruption of ER homeostasis (Klebanovych et al., 2022) and triggering an ER stress response. The accumulation of proteins that are either misfolded or not folded correctly leads to ER stress (Chen and Cubillos-Ruiz, 2021; Gonzalez-Gronow et al., 2021). When misfolded proteins continue to accumulate, they activate an adaptive response known as the UPR. The repercussions of the UPR include the suppression of protein biogenesis, the modulation of gene expression, and the determination of cellular outcomes (such as apoptosis) (Chen X. et al., 2023). The UPR is a cellular adaptive mechanism that enables cells to respond to toxic stress. Dysfunction of this mechanism is frequently observed in various pathological conditions associated with neurological disorders (Liu Z. et al., 2023). In eukaryotic cells, the UPR restores protein homeostasis by reprogramming gene transcription and protein modification to clear the burden of misfolded or unfolded proteins (Hetz et al., 2020). HSPA5 serves as a key regulator of the UPR by interacting with transmembrane ER stress sensors (Chen Y. et al., 2023). HSPA5 participates in ER stress through its substrate-binding domain, which can bind to misfolded or unfolded protein fragments. HSPA5 subsequently transmits the stress signal to inositol-requiring enzyme 1α (IRE1α) and protein kinase R-like ER kinase (PERK) via its ATPase domain, leading to the dissociation of the sensor–HSPA5 complex (Hetz et al., 2020). These observations suggest that HSPA5 also acts as a sensor for ER stress. Under conditions of protein homeostasis, BiP binds to these sensors (transcription factor 6 [ATF6], IRE1α and PERK) and maintains them in an inactive state (Li F. et al., 2022). When ER stress occurs, the binding affinity of HSPA5 for misfolded or unfolded proteins increases, and the protein thus dissociates from the sensors. The dissociation of HSPA5 allows the sensors to become activated, thereby inducing the UPR (Liu H. et al., 2020). The UPR is regulated by three transmembrane sensors located in the ER: ATF6, IRE1α, and PERK (Yang et al., 2021). ATF6 undergoes ER-to-Golgi translocation, whereupon its cytosolic moiety is subjected to sequential proteolysis mediated by site-1 protease (S1P) and site-2 protease (S2P), producing an active transcription factor that specifically binds to ER stress response elements in the promoter regions of target genes, thereby initiating the transcription of a series of genes (Díaz-Bulnes et al., 2020). Conversely, when activated, PERK forms dimers and adds a phosphate group to eukaryotic translation initiation factor 2 subunit α (eIF2α), which in turn suppresses the initiation of translation for most proteins (Xu et al., 2022). eIF2α phosphorylation specifically starts the translation process of activating transcription factor 4 (ATF4) (Oku et al., 2021). Upon activation, IRE1 oligomerizes and initiates the regulated IRE1-dependent degradation pathway (RIDD), which degrades ER-targeted mRNAs. This IRE1-mediated mRNA degradation reduces the influx of nascent polypeptides into the ER, thereby alleviating ER stress (Kim et al., 2021). When ER stress remains unresolved, the PERK and IRE1 pathways trigger the expression of the proapoptotic transcription factor C/EBP homolog (CHOP) and activate the JNK pathway, leading to apoptosis (Ahlstedt et al., 2024). When the UPR is activated, it reduces the overall rate of protein synthesis, increases lipogenesis (Ward et al., 2022), and induces the synthesis of ER lumen proteins that assist in folding, as well as the expression of ERAD system components. If these adjustments are not adequate for normalizing ER homeostasis, sustained activation of the UPR ultimately leads to programmed cell death (Radanović and Ernst, 2021).

### 2.4 HSPA5 and cellular autophagy and apoptosis

Through catabolic pathways, autophagy dismantles various components, including damaged organelles, pathogens, and misfolded proteins (Foerster et al., 2022; Jiao et al., 2023). If the stress from misfolded proteins remains unresolved, the UPR then initiates the autophagic degradation of sections of the ER, called ER autophagy (Elfiky et al., 2020). Autophagy serves as a crucial defense mechanism for cells under ER stress (Foerster et al., 2022). Autophagy triggered by ER stress can eliminate misfolded or unfolded proteins and damaged organelles. Evidence suggests that HSPA5 participates in triggering autophagy, thereby removing misfolded proteins in both soluble and aggregated forms, as well as dysfunctional organelles (Leiva-Rodríguez et al., 2021). HSPA5 promotes the recruitment of misfolded proteins and other molecules to autophagosomes by binding to SQSTM1/p62 and inducing a conformational change, leading to the eventual degradation of the misfolded proteins into amino acids. Therefore, HSPA5 mediates the clearance of misfolded proteins prone to aggregation via the macroautophagy pathway (Casas, 2017). Furthermore, HSPA5 expression increases under various ER stress conditions, contributing to enhanced cellular survival mechanisms. HSPA5 inhibits apoptosis and increases survival rates by blocking the interaction between caspase activation and the apoptotic pathway (Yuan et al., 2021). HSPA5 interacts with caspase-7 and inhibits its activation to prevent apoptosis, promote cell protection, and regulate chemoresistance. Conversely, inhibition of HSPA5 triggers the UPR and leads to caspase-4-mediated apoptosis (Samanta et al., 2021). However, if ER stress persists and triggers excessive autophagy, it results in massive organelle degradation and disruption of cellular homeostasis (Kawaguchi et al., 2020). When this occurs, ATF6, IRE1α, and PERK become activated, ultimately leading to cell death through CHOP-dependent apoptotic signaling (Mi et al., 2023). A close association exists between CHOP-induced apoptosis and ER redox enzyme 1α (ERO1α). After ERO1α activates inositol 1,4,5-triphosphate receptor 1 (IP3R1), it opens calcium channels on the ER membrane, releasing Ca^2+^ into the cytoplasm. Ca^2+^ in the cytoplasm subsequently activates calcium/calmodulin-dependent protein kinase II (CaMKII) and NADPH oxidase on the cell membrane. This mechanism leads to the massive production of reactive oxygen species (ROS), ultimately resulting in cell death (Mekahli et al., 2011). Mouse experiments have revealed that caspase-12 is localized to the ER membrane and plays a central role in the initiation of ER stress-induced apoptosis in mice (Kang et al., 2024). Its activation depends on the HSPA5–CHOP–ERO1α signaling pathway. Through this pathway, the ER releases Ca^2+^ into the cytoplasm, which in turn activates calpain. Activated calpain cleaves caspase-12, thereby activating it. Once activated, caspase-12 is transported from the ER to the cytoplasm, where it triggers other caspase effector proteins in the neuron, thereby mediating neuronal apoptosis (Wang et al., 2024; Zhang J. et al., 2022). Notably, in rodents, caspase-12 is a key mediator of ER stress-induced apoptosis. However, in humans, owing to the presence of a premature stop codon, it primarily produces a truncated, nonfunctional protein, and key residues in its active site have also undergone mutations. Therefore, human caspase-12 does not participate in ER stress-induced apoptosis, whereas caspase-4 is the human homolog involved in this process (Hitomi et al., 2004). ER stress is illustrated in Figure 1.

**FIGURE 1:**
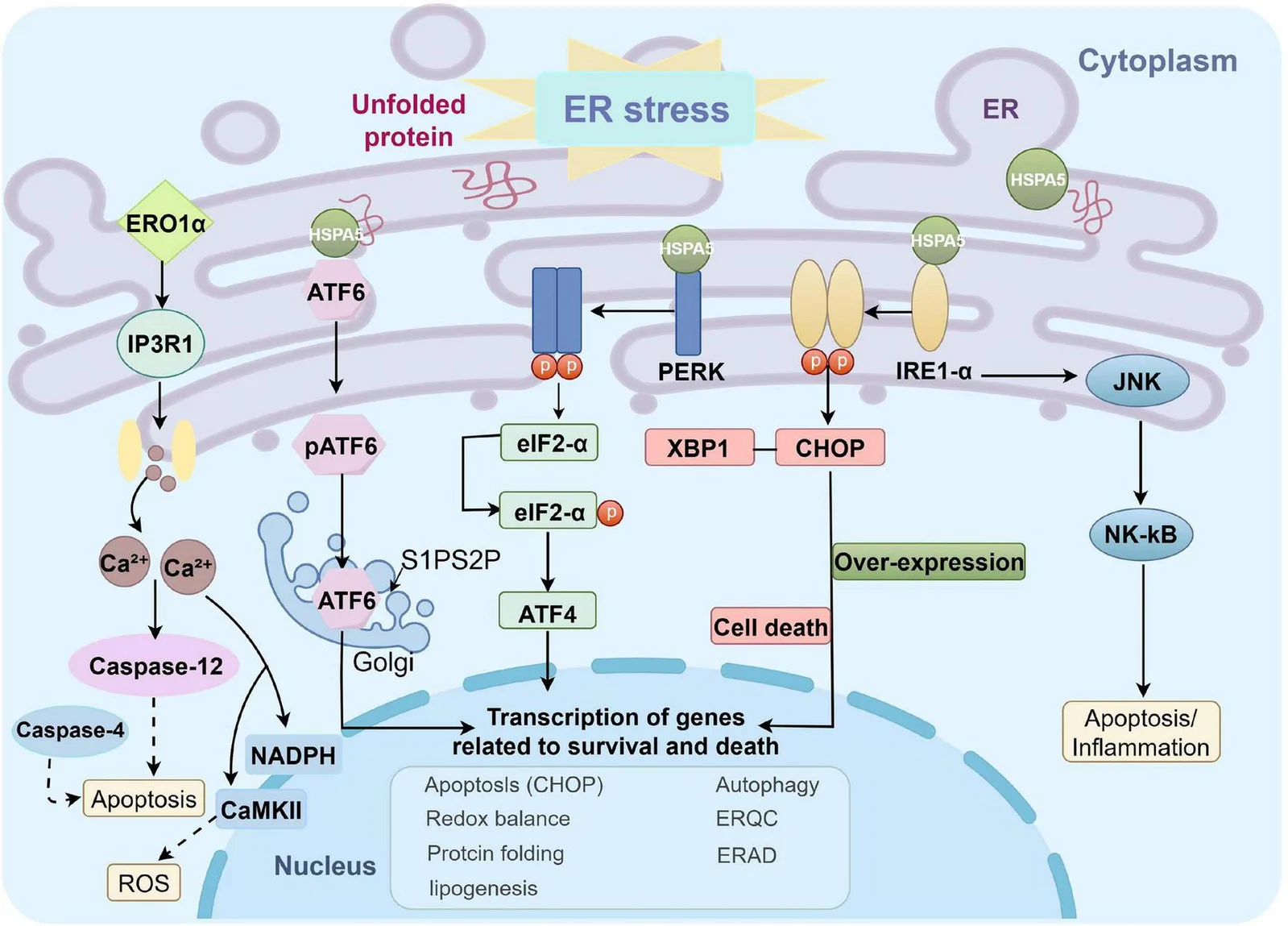
Schematic of the ER stress mechanism.

## 3 HSPA5 and environmental toxins

As the final link in the ecosystem, humans are inevitably exposed to various environmental toxins, and health risks are ubiquitous. The neurological damage caused by environmental toxins (such as PAHs, MPs, lead, cadmium, and arsenic) to the human body is shown in Table 1. HSPA5 is closely associated with environmental toxins; environmental toxins can induce ER stress by generating substantial quantities of ROS and triggering oxidative stress. During this process, HSPA5 is initially upregulated to provide adaptive protection, assisting in proper protein folding to maintain cellular homeostasis. However, prolonged or high-dose exposure to this toxin may cause unresolved stress in the ER, with HSPA5 overexpression subsequently activating the apoptotic pathway.

**TABLE 1:** Mechanisms of the effects of environmental toxins on the human body and their neurotoxic effects.

Environmental toxins	Main sources	Cellular mechanisms	Neurotoxicity	References
PAHs	Incomplete combustion of organic matter (vehicle exhaust, coal combustion, barbecuing, smoking)	BaP binds to the AHR receptor, affecting the expression of cytochrome P450 CYP1A1 and CYP1A2 and leading to oxidative stress	Astrocyte toxicity and increased risk of neurodegenerative diseases	Bukowska et al., 2022; Lu et al., 2024; Wang et al., 2025
Microplastics	Plastic degradation (cosmetics, textile washing, plastic waste)	Enzymatic reactions catalyzed by NADPH oxidase or other enzymes generate large amounts of ROS, resulting in the production of superoxide and hydrogen peroxide	Neurodevelopmental abnormalities, potential cognitive impairments, and behavioral deficits in rats	Prüst et al., 2020; Vojnits et al., 2025
Lead	Manufacture of leaded gasoline, lead-based paints, lead pipes, and solder	Cation simulation (Ca^2+^, Zn^2+^), oxidative stress, and thiol binding	Disruption of the release of neurotransmitters, leading to neuron loss and an increased risk of neurodegenerative diseases	Shvachiy et al., 2023a; Jomova et al., 2025
Cadmium	Fossil fuel combustion, mining, industrial emissions, the use of cadmium-containing fertilizers in agriculture, and tobacco smoking	Crossing the blood-brain barrier and accumulating; severe oxidative stress; Ca^2+^ metabolic disorders	The onset and progression of AD	Deng et al., 2024; Huang et al., 2026
Arsenic	Groundwater with high arsenic levels, coal combustion, and soil and water bodies in areas affected by industrial pollution	Metabolism-related oxidative stress upregulates HSPA5 expression and triggers the NLRP1 inflammasome	The onset of neurodegenerative diseases and damage to the centra and peripheral nervous systems	Capelo et al., 2022; Jeong et al., 2023; Li H. et al., 2022; Li X. et al., 2022

### 3.1 PAHs

PAHs, or polycyclic aromatic hydrocarbons, are organic substances that are frequently present in the environment. They are produced through the burning of fossil fuels, emissions from vehicles, and cigarette smoke (Tartaglione et al., 2023). PAHs can be generated through multiple processes and present significant risks to both the environment and human health (Wang et al., 2025). Extended contact with these pollutants may lead to various diseases, such as cancer and neurodegenerative conditions (Chakravarty et al., 2022). Benzo[a]pyrene (BaP) is a typical PAH that is significantly toxic and neurotoxic and can induce oxidative stress (Bukowska et al., 2022). When the human body absorbs BaP, it connects with the AHR receptor in the cytoplasm. The resulting complex is transported into the cell nucleus, where it forms an activated heterodimeric complex with the AHR nuclear transporter. This heterodimer upregulates the expression of phase I metabolic enzymes (such as cytochrome P450 CYP1A1 and CYP1A2), which catalyze the generation of ROS through oxidative metabolic reactions (Xu et al., 2024). Excessive ROS production exceeds the body’s ability to scavenge them, leading to redox imbalance, which disrupts the ER protein folding mechanism. This leads to the buildup of improperly folded proteins in the ER, which in turn initiates ER stress (Chen et al., 2022; Mohás-Cseh et al., 2022). Studies have demonstrated that the autophagy induced by BaP in human astrocytes is associated with increased expression of PERK, IRE1, and HSPA5, which serve as markers of ER stress. At low concentrations (1 to 10 μM), cells exhibited a consistently high survival rate after 48 h of exposure to BaP. When the cells were exposed to 50 μM BaP, the survival rate decreased slightly. Cells exposed to pyrene at concentrations ranging from 1–100 μM also exhibited consistently high survival rates. However, when exposed to concentrations ranging from 250–1,000 μM, the survival rate decreased to approximately 50%. These results suggest that at high concentrations, the synergistic effects of autophagy and ER stress induced by BaP result in significant toxicity to astrocytes (Siangcham et al., 2025). Notably, oxidative stress itself may also induce similar risks associated with ER stress components, and both are extensively involved in the pathological mechanisms of aging and Alzheimer’s disease (AD) (Bánhegyi et al., 2007). Protein oxidation can cause reversible or irreversible modifications, impairing protein activity, solubility, and stability (Fu et al., 2022). HSPA5 couples redox imbalance in the ER lumen with UPR activation through the sensing of misfolded proteins; this function plays a crucial role in acute, severe ER stress (Bánhegyi et al., 2007). Furthermore, large insoluble aggregates, amyloid proteins, and certain denatured proteins activate the ability of the HSP70 system to disaggregate abnormally aggregated proteins. In its ATP-bound state, HSP70 recognizes protein aggregates with low affinity, whereas upon binding ADP, it transitions to a high-affinity state. Furthermore, HSP70 responds to oxidative stress by mediating the disassembly and reassembly of the 26S proteasome (Niforou et al., 2014). These findings further highlight the role of the HSP70 system in maintaining proteome stability. However, large amounts of benzo[a]pyrene-7,8-diol-9,10-epoxide (BPDE) can decrease cell viability, change the morphological structure of the ER, and greatly increase the expression levels of HSPA5 and p-PERK, causing ER stress and encouraging apoptosis (Zhang Q. et al., 2022).

### 3.2 MPs

MPs are a new type of pollutant and are plastic particles with a diameter of less than 5 millimeters. These materials remain in the environment for extended durations, and as global emissions increase sharply annually, their potential risks to human health have become increasingly concerning (Zhao B. et al., 2024). Living organisms can take in MPs through inhalation, eating, and consuming water (Bai et al., 2024; Cox et al., 2019). Research estimates indicate that the total annual intake of MPs by an individual via the digestive and respiratory tracts is approximately 74,000–121,000 particles (Sobhani et al., 2020). Furthermore, MPs can accumulate in multiple organs, inducing oxidative stress, which in turn leads to tissue structural damage and functional impairment, ultimately disrupting normal physiological processes in the organism (Bai et al., 2024). MPs can be internalized by cells via endocytosis or pinocytosis (Wang et al., 2020). Upon entering phagocytes, MPs trigger innate immune defense responses and are identified as potentially infectious foreign entities. When cells try to counteract these potentially infectious foreign entities, they generate large amounts of ROS through enzymatic reactions facilitated by NADPH oxidase or other enzymes, resulting in the production of superoxide and hydrogen peroxide (Hu and Palić, 2020). ROS are key triggers of ER stress, both of which lead to ferroptosis and cell death. Nanoplastic particles trigger cell death by inducing apoptosis and ferroptosis via ROS-mediated ER stress pathways (Wu Q. et al., 2024). Among these factors, the concentration and duration of exposure to MPs are particularly critical; studies have demonstrated that high concentrations of MPs (50 mg/kg and 100 mg/kg) administered continuously to pregnant mice for 14 days can significantly induce oxidative stress. The HSPA5/IRE1α/JNK pathway is directly activated by ROS, thereby significantly triggering ER stress (Bai et al., 2024). In addition, exposure to polystyrene MPs leads to a significant increase in ROS levels in the testicular interstitial cells of mice; this process drives apoptosis by upregulating the expression of proapoptotic regulators, ultimately resulting in an imbalance in ER homeostasis (Zhao et al., 2025). In female rats, exposure during pregnancy and lactation can induce sex-specific neurodevelopmental abnormalities and long-term cognitive deficits in their offspring (Vojnits et al., 2025). Furthermore, MPs may impair learning and memory by inducing ER stress. Using miRNAs to decrease the levels of HSPA5, PERK, and CHOP successfully mitigates ER stress and enhances cognitive functions (Mohammadi et al., 2025). The above findings suggest that environmental toxins can activate the HSPA5/UPR axis; however, this evidence is derived from nonneuronal models, and its applicability to human neurons remains to be verified. As terminally differentiated cells, neurons may exhibit UPR regulatory characteristics that differ from those of proliferating or peripheral cells; therefore, the direct extrapolation of these mechanisms should be regarded as a scientific hypothesis rather than an established conclusion, and further validation is urgently needed.

### 3.3 Heavy metals

Heavy metals have attracted widespread attention as harmful environmental pollutants due to the health risks they pose. The heavy metals lead, cadmium, and arsenic are widely found in different environmental media, such as air, water, soil, food, and industrial products, posing ongoing risks to human health (Pan et al., 2024). Recent studies have demonstrated that ER stress is a key mechanism through which these heavy metals cause cellular damage and that there is a close association between neurological disorders and exposure to toxic heavy metals (Ghosh et al., 2026; Xiao et al., 2022).

#### 3.3.1 Lead

Lead (Pb) is considered a Category 1 threat to both the environment and human health. Humans are especially vulnerable to lead exposure from industrial uses, such as the manufacturing of leaded gasoline, lead-containing paints, lead pipes, and solder (Liu X. et al., 2023). Lead poisoning involves mainly oxidative stress, disruptions in calcium balance, and changes in ER stability. These mechanisms can all be indirectly triggered by the interaction of lead with glutathione and antioxidant enzymes (Abdelrazek et al., 2022; Klinova et al., 2022; Liu Z. H. et al., 2020). Lead reduces the activity of enzymes that scavenge ROS and increases ROS production. It also promotes lipid peroxidation, thereby causing oxidative stress-related damage to the body (Kou et al., 2020). Protein folding depends on redox balance. When oxidative stress disrupts protein folding, it increases the load on the ER. In addition, lead poisoning can cause ER swelling, dilation, and vacuolization. When ER stress occurs, HSPA5 is released from these complexes to assist in the proper folding of accumulated proteins (Li N. et al., 2021). The Pb concentration is positively correlated with the degree of autophagy and ER stress induction. Specifically, Pb induces autophagy by inhibiting mTOR phosphorylation and p62 protein levels, upregulating the LC3-II/I ratio, and increasing the expression of autophagy-related genes; moreover, the intensification of ER stress manifests as elevated HSPA5 levels and enhanced splicing of XBP1. Studies have demonstrated that under mild Pb exposure (0–100 μM), autophagy and ER stress act as adaptive regulatory mechanisms; however, under severe Pb exposure (500 μM), the excessive activation of these two mechanisms induces apoptosis, ultimately threatening cell survival (Liu et al., 2026). Studies using rat models have demonstrated that when rats are exposed to lead from the embryonic stage through old age, dynamic changes in neurodegeneration, oxidative stress, and ER stress are observed in their brains. In PNW3 and PNW70 rats, following exposure to Pb, HSPA5 expression increased, and the neuronal density and brain volume decreased. Lifelong exposure to Pb induces neurodegenerative damage, which begins in infancy, subsides somewhat in adulthood, but worsens in old age. In astrocytes, Pb interferes with the function of HSPA5 by directly binding to it, preventing it from effectively assisting in the proper folding of accumulated proteins. Moreover, the levels of ER stress signaling pathway components, total JNK and phosphorylated JNK, markedly increase, which is accompanied by an increase in the expression of the apoptosis marker CHOP and a decrease in the expression of the antiapoptotic protein BCL-2 (Shan et al., 2024). These findings indicate that Pb can bind to and upregulate the expression of HSPA5. This prevents HSPA5 from performing its original adaptive function, and the ER stress response is redirected toward the apoptotic pathway.

#### 3.3.2 Cadmium

Cadmium (Cd) is a heavy metal known for its high solubility in water and originates from fossil fuel combustion, mining activities, and agricultural fertilizer use (Li R. et al., 2025). Moreover, Cd can cross the blood–brain barrier and accumulating in the brain, leading to neurotoxic effects. Cd exposure is a potential environmental risk factor that contributes to the progression of AD. Multiple studies have demonstrated that blood Cd levels are significantly higher in patients with AD than in healthy controls (Deng et al., 2024). Extended contact with Cd may lead to oxidative stress, causing persistent damage to the ER and ultimately resulting in apoptosis (Wu J. et al., 2024). Cd is located mainly in the ER within cells, and its accumulation causes structural and functional damage to this organelle (Rana, 2020). This toxic effect triggers ER stress, which ultimately initiates the process of programmed cell death (Zhang et al., 2024). Cells exposed to Cd exhibited a dose-dependent increase in HSPA5 expression. Studies have demonstrated that among the treatment groups exposed to Cd concentrations of 0, 1, 2, and 4 mg/L, the ROS levels in the 4 mg/L Cd group were significantly greater than those in the 1 and 2 mg/L Cd groups; furthermore, compared with 0 mg/L Cd, 4 mg/L Cd significantly reduced cell viability. Moreover, the protein levels of HSPA5 and ATF6 were significantly elevated (Liu J. et al., 2025). Activation of the Cch1/MID1 calcium channel by HSPA5 promotes Cd influx, causing an imbalance in intracellular calcium levels. This mechanism implies that Cd triggers ER stress by increasing HSPA5 expression and altering calcium equilibrium (Rana, 2020). Consequently, mitigating continuous stress in the ER and restoring its equilibrium could be a method to lessen the cytotoxicity induced by Cd.

#### 3.3.3 Arsenic

Inorganic arsenic (As) can be found in environmental elements such as soil, rock, water, and air. Worldwide, approximately 220 million individuals face a significant risk from consuming water heavily tainted with arsenic (Virolainen et al., 2023). The U.S. Agency for Toxic Substances and Disease Registry classifies arsenic as one of the most dangerous substances, posing an ongoing risk to human health (Li J. et al., 2025). The atmosphere accumulates significant amounts of arsenic due to coal burning and industrial wastewater release. Inhalation is the primary route of exposure to arsenic (Zhao et al., 2022). Acute exposure to high concentrations of arsenic can damage the central and peripheral nervous systems. Chronic exposure can cause lasting damage to multiple organs, including the gastrointestinal, respiratory, and central nervous systems, and may even lead to cancer (Capelo et al., 2022). Recent studies have verified that ER stress plays a role in the neurotoxicity caused by arsenic and is strongly associated with the onset of neurodegenerative diseases related to arsenic exposure (Wadgaonkar and Chen, 2021). HSPA5 expression is regulated at both the transcriptional and posttranscriptional levels. In human bronchial epithelial cells, As2O3 increases the phosphorylation levels of eIF2α and IRE1α in a dose-dependent manner and upregulates the expression of cleaved ATF6α (Yuan et al., 2021). An internal ribosomal entry site (IRES) found before the start codon of HSPA5 mRNA might facilitate the enhancement of its translation efficiency. Furthermore, the activation of the IRE1α ribonuclease promotes the rapid degradation of certain microRNAs that inhibit mRNA translation, thereby increasing the suppression of HSPA5 translation (Cho et al., 2007). Consequently, arsenic trioxide (As2O3) can significantly enhance the biosynthetic efficiency of the HSPA5 protein (Yuan et al., 2021). These findings indicate that As2O3 upregulates HSPA5 expression at both the transcriptional and translational levels and can activate the ER stress signaling pathway. HSPA5 is hypothesized to play a role in this process. During ER stress, As2O3 triggers the leucine-rich repeat and pyridine-containing domain 1 (NLRP1) inflammasome, which mediates inflammatory responses and apoptosis. The NLRP1 inflammasome is a key component of cell death mechanisms. Overexpression of NLRP1 may lead to neuronal death (Chu et al., 2025). Notably, although the NLRP1 inflammasome is considered a key molecule in neuronal cell death, its functional relationship with the HSPA5/UPR axis under As2O3 exposure remains to be elucidated.

## 4 Impact of HSPA5 on neurodegenerative conditions

Environmental toxins are among the primary factors contributing to neurological diseases; long-term exposure to environmental toxins can accelerate the progression of neurodegenerative diseases and trigger their rapid development (Newell et al., 2025). HSPA5 is essential for understanding the development of neurodegenerative diseases. The role of HSPA5 in neurodegenerative diseases is illustrated in Figure 2 and Table 2. During the initial phases, it plays a vital protective role by ensuring protein balance, acting as the first line of defense in the neuronal response to ER stress. In the later phases of the disease, its function becomes disrupted or irregular, intensifying the apoptotic signals of the UPR and advancing neurodegenerative pathological processes (Singh R. et al., 2024).

**TABLE 2:** HSPA5/UPR signaling in response to environmental stressors and its association with neuronal dysfunction in experimental models.

Disease	Stressor	UPR pathway	Experimental model	Neuron results	Changes and functions of HSPA5	References
PD	Lead	α-syn overexpression and complex formation with HSPA5	Rat model	Death of dopaminergic neurons	Overexpression reduced the death of dopaminergic neurons	Shvachiy et al., 2023b; Li et al., 2024
AD	Environmental pollutants	Upregulation of HSPA5, PERK, IRE1α, PERK, p-eIF2α, and CHOP	Brain tissue from patients with AD obtained at autopsy	Synaptic dysfunction and neuronal death	Significantly increases and reduces Aβ production and promotes the correct folding of APP	Singh R. et al., 2023; Dhapola et al., 2024; Ekundayo et al., 2024
HD	Cd	Elevated levels of HSPA5, CHOP, and P-PERK proteins; phosphorylation of eIF2α	Mouse model	Inhibition of neuronal plasticity	Reduced expression prevents the loss of dendritic spines in hippocampal neurons	Espina et al., 2023; Sahoo and Sharma, 2023
ALS	BaP	HSPA5 expression was significantly elevated	Fruit fly model	Degeneration of motor neurons	Upregulation of HSPA5 alleviates TDP-43-induced toxicity and promotes its proper folding	Xu et al., 2025; François-Moutal et al., 2022

**FIGURE 2:**
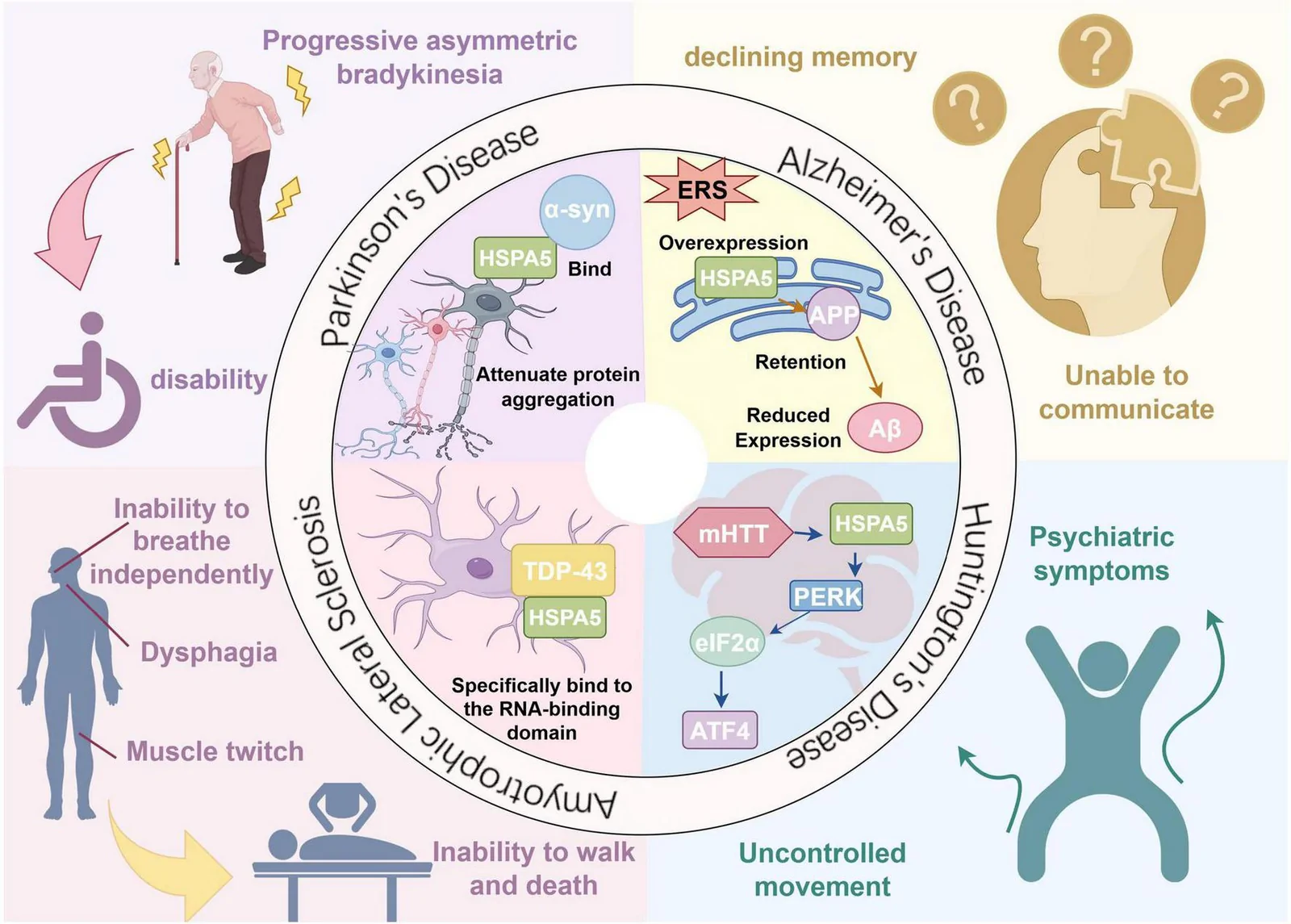
Diagram of the role of HSPA5 in neurodegenerative diseases.

### 4.1 Role of HSPA5 in Parkinson’s disease (PD)

The prevalence of PD, a neurodegenerative disorder, is increasing quickly (Langeskov-Christensen et al., 2024). PD is often characterized by uneven slowness of movement, tremors, and walking difficulties; in extreme cases, it can result in disability and cognitive decline. The burden of this disease has become a worldwide public health concern (Morris et al., 2024). The onset of PD is closely linked to environmental factors. As pollutants, particulate matter and heavy metals can modify oxidative stress pathways in certain areas of the brain and nervous system (Aravindan et al., 2024). PD has a varied pathological foundation, involving elements such as tauopathy, AD-related pathological changes, and the selective loss of nigral neurons without the usual protein aggregates (Han et al., 2021; Morris et al., 2024; Trinh et al., 2021). PD is strongly linked to harm to neurons in the substantia nigra, where the loss of dopaminergic neurons results mainly from the intricate interaction between oxidative stress and faulty protein degradation processes (Hill et al., 2023). This damage is characterized by the formation of Lewy bodies, which are aggregates of α-synuclein (α-syn) (Horsager and Borghammer, 2024). α-Syn is a protein found at very high concentrations in neurons; it is localized primarily at synaptic terminals and plays a role in regulating neurotransmitter release. Under physiological conditions, it exists in dynamic equilibrium, switching between a soluble, unfolded state and a structure containing amphipathic α-helices (which mediate binding to membrane lipids) (Ye et al., 2023). In animal models, lead exposure leads to increased α-syn levels (Shvachiy et al., 2023b). α-Syn inhibits the function of HSPA5 by directly forming a complex with it, thereby exacerbating ER stress. In models of PD, both in cells and in animals, where α-syn is overexpressed, the UPR pathway remains persistently activated. Remarkably, in a rat model of PD, the UPR signaling pathway was modulated by the overexpression of the HSPA5 gene, which significantly reduced dopaminergic neuron death and enhanced their survival (Li et al., 2024). This mechanism plays a significant role in protein toxicity in PD, and regulating HSPA5 can effectively inhibit the formation of abnormal protein aggregates. Furthermore, studies have demonstrated that HSPA5 overexpression has a significant neuroprotective effect on dopaminergic neurons through the maintenance of protein homeostasis, and intranasal administration of HSPA5 alleviated neurodegenerative processes in a rat model simulating the preclinical stage of PD (Casas, 2017; Pazi et al., 2024). The pathological aggregation of α-syn is a heterogeneous, multistep process. Under normal conditions, α-syn exists as soluble, unfolded monomers, a conformation that serves as the starting point for aggregation (Levin et al., 2022; Li N. et al., 2021). When continuously exposed to environmental toxins and oxidative stress, α-syn misfolds, mainly taking on a β-sheet structure. Initially, α-syn forms soluble oligomers that later develop into insoluble protofibrils. Ultimately, these fibers form Lewy bodies within the dopaminergic neurons of the substantia nigra pars compacta, which leads to permanent neuronal damage and degeneration (Salim et al., 2023). Research has indicated that the UPR is significantly activated in the substantia nigra of individuals with PD and that this activation level is positively linked to the amount of α-syn aggregation (Cheng et al., 2023; Jiang J. et al., 2022).

### 4.2 Role of HSPA5 in AD

AD is a disorder that progressively worsens and degenerates, impacting the nervous system (Khan et al., 2020). AD is gradually becoming the neurodegenerative disease with the highest mortality rate and the most far-reaching social impact of this century (Scheltens et al., 2021). Long-term exposure to environmental toxins exacerbates neuropathological lesions, thereby contributing to the onset of AD. Common mechanisms of the toxic effects of environmental pollutants include oxidative stress, tau protein abnormalities, and abnormal handling of the amyloid precursor protein (APP) (Dhapola et al., 2024). AD is characterized primarily by the abnormal buildup of extracellular β-amyloid (Aβ) plaques, the development of neurofibrillary tangles (NFTs) composed of hyperphosphorylated tau within neurons, and the subsequent effects (Zheng and Wang, 2025). Aβ is derived from APP (Singh A. et al., 2023), and the pathogenesis of AD involves impaired metabolic processing of APP. During its translocation from the ER to the Golgi complex, APP interacts with various molecular chaperones, including HSPA5, GRP94, and CHIP (Singh R. et al., 2023). Specifically, under ER stress conditions, overexpression of HSPA5 retains APP in early secretory compartments and interacts with E3 ubiquitin ligase (Pirh2), thereby inhibiting Aβ aggregation and reducing Aβ production (Casas, 2017; Singh A. et al., 2023). Therefore, this molecular chaperone system jointly manages and facilitates the maturation of intracellular APP, enhancing its correct folding (Singh R. et al., 2023). Oligomeric forms of Aβ are key drivers in the pathogenesis of AD; they participate in the neurodegenerative process of AD by inducing ER stress in neurons, which in turn activates downstream pathways such as the UPR (Yoo and Joo, 2023). Toxic soluble pTau, Aβ oligomers, and NFTs can inhibit ERAD and proteasome functions. This leads to an increased buildup of proteins in the ER, which then activates the UPR (Shacham et al., 2021). Sustained activation of the UPR triggered by ER stress is one of the core mechanisms underlying fundamental synaptic dysfunction and neuronal death (Yasmeen et al., 2022). HSPA5 expression is abnormally elevated in pathologically affected regions of the brain tissue of patients with AD (Singh R. et al., 2023). Postmortem brain tissue from patients with AD exhibits marked upregulation of the expression of canonical ER stress markers, including HSPA5, UPR sensors (PERK and IRE1α), the PERK-specific downstream effector p-eIF2α, and the apoptotic mediator CHOP. Research implies that the continuous triggering of the ER stress response influences the progression of neurodegeneration in AD (Ekundayo et al., 2024). The data indicate that HSPA5 expression is significantly elevated in the frontal cortex, hippocampus and temporal cortex of patients with AD. Furthermore, HSPA5 levels are increased in hippocampal tissue lysates and within the neuronal milieu of postmortem AD brains. Importantly, HSPA5 levels correlate positively with the Braak staging system (based on NFTs and amyloid plaque deposition), which reflects disease severity, suggesting that its expression increases as AD progresses (Voronin et al., 2023).

### 4.3 Influence of HSPA5 in Huntington’s disease (HD)

HD is an autosomal-dominant neurodegenerative condition (Barry et al., 2022). Typically, individuals experience a symptom-free period that lasts several decades before symptoms appear, followed by uncontrolled movements, cognitive impairment, and psychiatric symptoms. Eventually, motor symptoms progress to severe functional impairment and muscle rigidity, leading to death (Handsaker et al., 2025). HD is an autosomal-dominant disorder (Saade and Mestre, 2024). It is currently widely accepted that the disease is caused primarily by abnormal changes in the expression of the mutant huntingtin protein (mHTT) (Islam et al., 2023). The disease mechanism includes an abnormal increase in a trinucleotide repeat sequence (CAG) within exon 1 of the huntingtin (HTT) gene (Saade and Mestre, 2024). This sequence encodes an extended polyglutamine (polyQ) chain in the N-terminal region of the mHTT protein. This expansion causes the mHTT protein to misfold and aggregate abnormally within brain cells. The expression of this protein progressively disrupts neuronal physiological function (Tabrizi et al., 2022). mHTT oligomers induce ER stress by suppressing ERAD, ultimately leading to the activation of the UPR (Maity et al., 2022). In HD, soluble mHTT oligomers are key drivers of ER stress, primarily by activating the PERK pathway of the UPR (Shacham et al., 2021). In R6/1 mice during the early stages of the disease, sustained ER stress activation in the hippocampus manifested as elevated levels of the HSPA5, CHOP, and P-PERK proteins. No significant changes in HSPA5 or CHOP protein levels were detected in the striatum or cortex. However, in the very late stages of the disease, HSPA5 levels were significantly increased in the striatal cortex of R6/1 mice. Therefore, as a potential mechanism underlying HD-related hippocampus-dependent cognitive impairment, focusing on the unfolded protein response pathway is crucial. Continuous activation of PERK negatively affects cognitive abilities by chronically inhibiting the synthesis of key new proteins required for neuronal plasticity and memory formation (Hafycz et al., 2022; Shacham et al., 2021). Mice with elevated HSPA5 expression exhibited increased PERK phosphorylation levels in the hippocampus, whereas no significant changes were observed in the phosphorylation levels of IRE1α, a downstream effector of UPR sensor proteins. These findings suggest that the HSPA5/PERK axis is specifically activated in the mouse hippocampus (Espina et al., 2023). PERK activation induces the phosphorylation of eIF2α (Cheng et al., 2021; Elvira et al., 2020). Phosphorylation of eIF2α leads to widespread and transient inhibition of protein translation, thereby alleviating protein overload and stress in the ER (Gorbatyuk et al., 2020). However, if stress persists, it can reduce the synthesis of key new proteins required for memory formation. However, studies have demonstrated that PERK inhibition does indeed reverse cognitive impairments in R6/1 mice, but this effect is not associated with reduced eIF2α phosphorylation. These findings suggest that other molecular pathways downstream of PERK and independent of eIF2α may contribute to memory deficits in HD. Furthermore, reducing the expression of HSPA5 decreases mHTT aggregation and reduces pathological changes in HD in the hippocampus (Espina et al., 2023).

### 4.4 Contribution of HSPA5 to amyotrophic lateral sclerosis (ALS)

The central nervous system is affected by the neurodegenerative disease known as ALS. Because the disease is difficult to recognize in its early stages, it can lead to diagnostic delays (Feldman et al., 2022). ALS affects both upper motor neurons (UMNs) and lower motor neurons (LMNs), impacting the medulla oblongata and the spinal cord. This causes a gradual decline in the strength of the voluntary muscles responsible for breathing, swallowing difficulties, and problems with limb movement and speech (Goutman et al., 2022). Environmental pollutants are broadly acknowledged as significant risk factors for ALS; exposure to environmental factors increases the likelihood of developing ALS and affects the progression of the disease through neurotoxicity and oxidative stress mechanisms (Xu et al., 2025). The neuropathological sine qua non of ALS is the cytosolic accrual and nuclear diminution of TDP-43, which manifests in > 97% of cases (Brown et al., 2022). TDP-43 is the primary protein that aggregates in patients with ALS (Trist et al., 2022). A commonly expressed RNA-binding protein (RBP) that is essential for RNA processing. Affected cerebral territories in patients with ALS exhibit a loss of physiological diffuse intranuclear TDP-43 distribution (Gruijs da Silva et al., 2022). In most patients with ALS, TDP-43 is incorrectly transported from the nucleus to the cytoplasm in affected neurons and glial cells, where it misfolds and forms aggregates (Konopka and Atkin, 2022). BaP induces ALS-associated TDP-43 expression and abnormal aggregation by activating the AhR signaling pathway. This process accelerates the degeneration of motor neurons (Xu et al., 2025). Studies in a Drosophila model have demonstrated that upregulation of HSPA5 expression can attenuate TDP-43-induced toxicity (François-Moutal et al., 2022). HSPA5 selectively engages the TDP-43 RNA-binding moiety. In the prefrontal cortices of patients with ALS, the expression of cytoplasmic HSPA5 is significantly elevated (Wang et al., 2015). Under oxidative stress conditions, HSPA5 interacts directly with TDP-43 (Feneberg et al., 2020). Evidence suggests that HSPA5 binds to TDP-43 in a non-RNA-binding conformation, ensuring its proper folding and preventing misfolding. Notably, although overexpression of Hsp70 can prevent the formation of TDP-43 aggregates, it cannot degrade or dissolve already-formed inclusions (François-Moutal et al., 2022). These results indicate that HSPA5 plays a crucial role in the onset and progression of ALS.

## 5 Outlook

In recent years, experts in many fields have speculated that ER stress may activate specific mechanisms that promote the relocalization of GRP78 and other ER chaperones to the cell surface, thereby extending cellular functions beyond the ER (Lee, 2025). This possibility suggests that the function of HSPA5 may extend far beyond its role as a molecular chaperone within the ER lumen. Extracellular HSPA5 (including secreted and soluble forms) may be involved in intercellular communication within the nervous system and play important roles in the interactions between neurons and glial cells (such as oligodendrocytes and astrocytes). Research by Miyamoto et al. revealed that extracellular HSPA5 interacts with the Plexin-B3 receptor, which is specifically expressed on the surface of oligodendrocytes, to regulate oligodendrocyte maturation and myelination (Miyamoto et al., 2026). Currently, in a rat model of Parkinson’s disease (PD), intranasal administration of exogenous HSPA5 exerts a neuroprotective effect by inhibiting α-syn pathology, blocking ER stress-induced apoptosis, downregulating endoplasmic reticulum stress mediators in the PERK-dependent pathway of the UPR, and preventing the activation of proapoptotic caspases-9 and caspases-3 (Pazi et al., 2024). This is illustrated in Figure 3. These findings suggest that extracellular HSPA5 may serve as a mediator of neuron-glial communication, opening up promising avenues for research into therapeutic biologics. They provide a more comprehensive perspective on the biology of HSPA5 and offer a new direction for understanding its role in neurological disorders.

**FIGURE 3:**
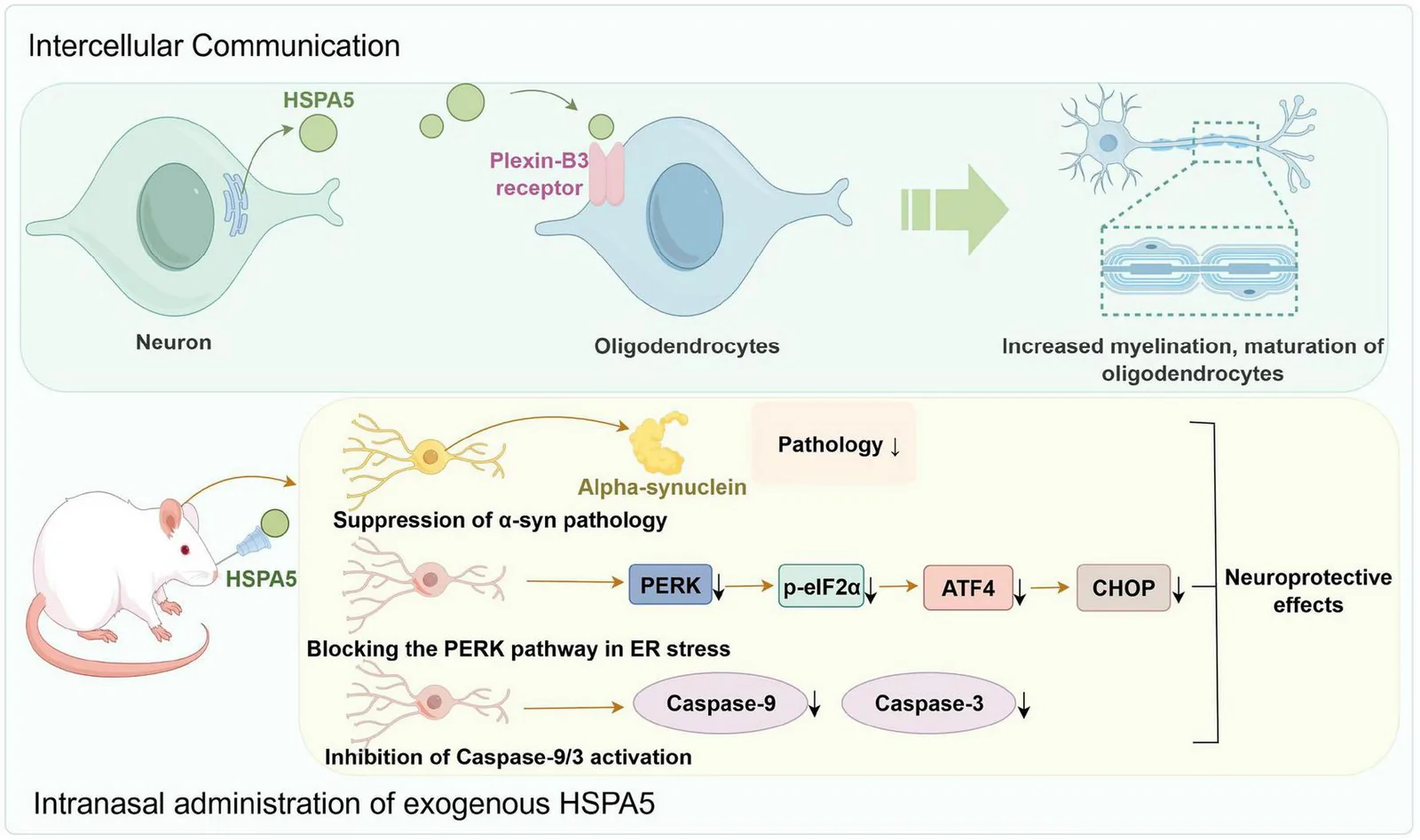
Extracellular HSPA5 is involved in intercellular communication in the nervous system.

## 6 Conclusion

In the ER, HSPA5 serves as an essential chaperone with a key function. It utilizes energy generated by ATP hydrolysis to assist these polypeptide chains in folding correctly and forming functional three-dimensional structures. In addition, it prevents protein misfolding, inhibits nonspecific aggregation among unfolded or partially folded proteins, and prevents the formation of harmful aggregates. HSPA5 plays a role in the cellular response to ER stress. When a cell initiates the adaptive UPR response, as the accumulation of misfolded proteins increases, HSPA5 preferentially interacts with these misfolded proteins, releasing three sensors. The released PERK and IRE1α are activated, initiating signaling pathways. Ultimately, this halts translation throughout the cell, reduces the synthesis of new proteins, and alleviates the burden on the ER. This process also upregulates the expression of chaperone molecules, including the massive synthesis of HSPA5 itself, thereby enhancing the protein folding capacity of the ER. Once released, ATF6 is transported to the Golgi, where it is cleaved; its cytoplasmic fragment is translocated to the nucleus, where it directly activates the genes that encode chaperones and ERAD components, which are subsequently expressed. In summary, HSPA5 senses stress levels and precisely regulates the intensity of the UPR through a “dissociation–activation” mechanism, thereby attempting to restore the consistency of the environment within the cell.

Environmental toxins such as PAHs, MPs, and heavy metals can stimulate cells to produce substantial quantities of ROS, leading to oxidative stress. The ER is highly sensitive to oxidative environments; oxidative stress disrupts its redox balance and inhibits the proper formation of disulfide bonds, thereby causing protein misfolding. In various animal models, a common feature of neurodegenerative diseases such as AD, PD, and HD is the aggregation of misfolded proteins. When cells are chronically exposed to environmental toxins beyond their compensatory capacity, ER stress cannot be resolved. Increased levels of ER stress and UPR signaling proteins contribute to the onset and progression of neurodegenerative diseases, and HSPA5 expression is upregulated in various neuropathological states. CHOP, a transcription factor involved in the apoptotic phase of the UPR, promotes apoptosis upon excessive activation. Therefore, it has been proposed that when environmental toxins cause abnormal protein aggregation and induce a persistent state of ER stress, this may impair HSPA5 function and shift the UPR from a pro-survival state to a pro-apoptotic state. Figure 4 illustrates this change. This, in turn, accelerates neuronal death and ultimately leads to the exacerbation of neurodegeneration. CHOP can serve as a biomarker for monitoring the apoptotic phase of the UPR.

**FIGURE 4:**
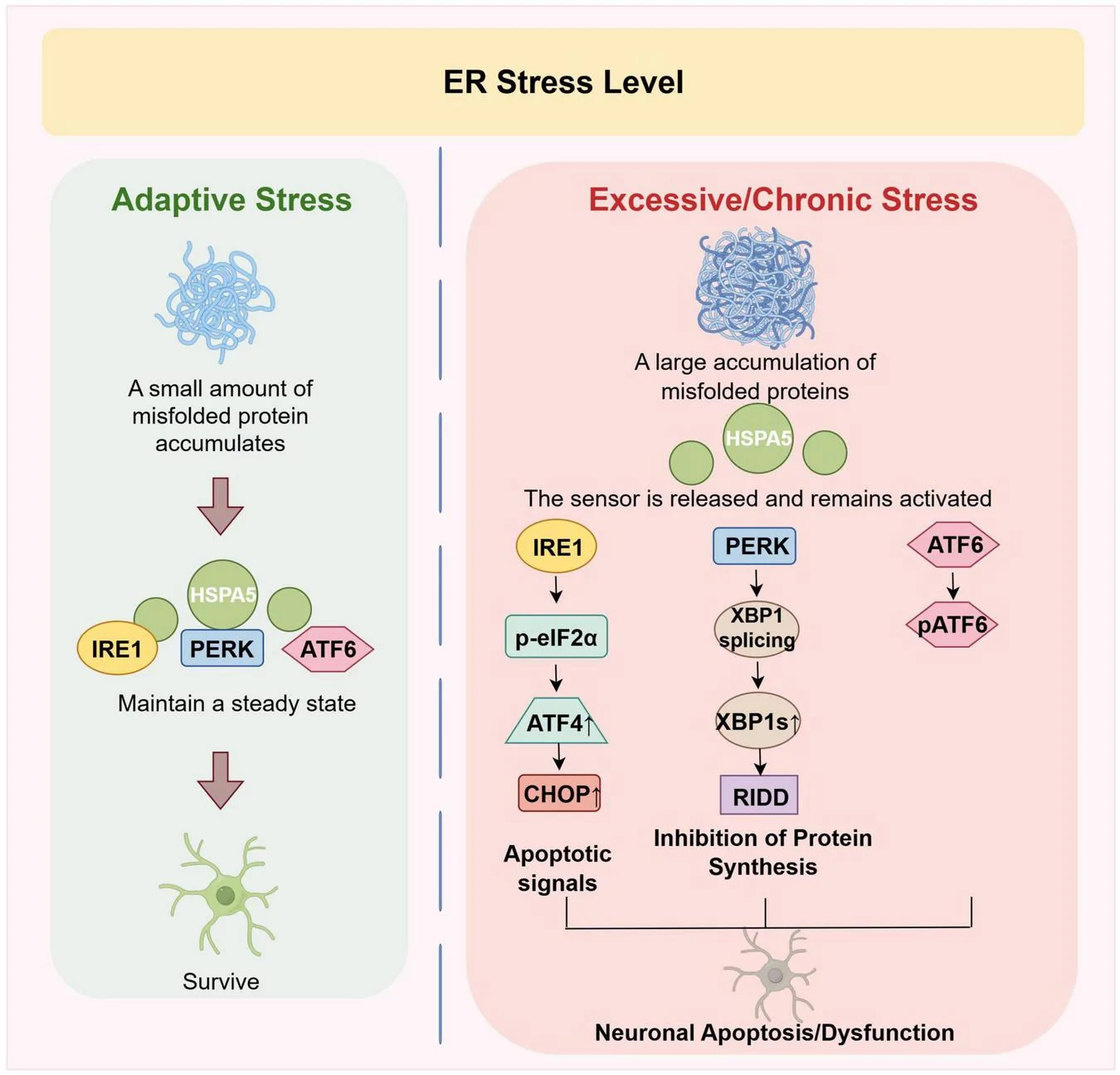
The dual role of HSPA5 in adaptation and under conditions of excessive endoplasmic reticulum stress.

Recent studies have reported that compared with those in healthy individuals, HSPA5 levels are elevated in patients with neurodegenerative diseases. Measuring HSPA5 levels in the cerebrospinal fluid or peripheral blood of individuals exposed to environmental factors is considered a potential marker for assessing neurotoxic damage. However, whether this method can serve as a reliable risk assessment tool or a biomarker for the early diagnosis of neurological dysfunction requires further validation in large-scale human cohort studies, particularly regarding its specificity for brain-derived ER stress. Although there is currently a lack of direct clinical evidence linking the regulation of HSPA5 expression to improved prognosis of neurodegenerative diseases, elucidating the regulatory mechanisms of the HSPA5/UPR signaling axis remains a critical area of research for understanding how environmental stressors lead to neuropathological changes. However, before proceeding, we must clarify that while HSPA5 is vital for environmental toxin-induced damage and neurodegenerative diseases—serving as the first line of defense in the cellular defense system—the ultimate failure of its function is also a key factor leading to irreversible neuronal damage. Many questions remain unresolved. For example, much of the evidence stems from nonneuronal cell models, whereas the activation patterns, signaling preferences, and ultimate outcomes (survival or apoptosis) of ER stress are cell type dependent. Its direct applicability in neurons remains unclear; therefore, studying these mechanisms in rodent and human brain tissues and neuronal cells is necessary. Other questions include how to distinguish between the protective and pro-apoptotic effects of HSPA5 and the UPR, as well as how to prevent excessive UPR activation and the transition to a pro-apoptotic state by regulating HSPA5 levels, among others. These are the key areas we need to address in future research.
